# Analyzing Power Beacon Assisted Transmission with Imperfect CSI in Wireless Powered Sensor Networks

**DOI:** 10.3390/s19040882

**Published:** 2019-02-20

**Authors:** Xuanxuan Tang, Wendong Yang, Yueming Cai, Weiwei Yang

**Affiliations:** College of Communications Engineering, Army Engineering University of PLA, No. 88 Houbiaoying, Qinhuai District, Nanjing 210007, China; tang_xx@126.com (X.T.); caiym@vip.sina.com (Y.C.); wwyang1981@163.com (W.Y.)

**Keywords:** energy harvesting, wireless sensor network, energy storage, power optimization, ergodic capacity

## Abstract

This paper proposes the maximal ratio transmission (MRT) and maximal ratio combining (MRC) protocols for the power beacon (PB) assisted wireless powered sensor networks and analyzes the impact of the imperfect channel state information (CSI) on the performance using the Markov chain theory. The wireless powered sensor chooses to transmit information to the destination or harvest energy from the PB when its energy can or cannot supply a transmission, respectively. The energy arrival and departure of the sensor is characterized, and the analytical expressions of the network transmit probability, and effective and overall ergodic capacities are formulated and derived. We also optimize the sensor transmit power to maximize the overall ergodic capacity. Our results reveal that the transmit probability and the effective ergodic capacity can be greatly improved with increasing the number of antennas at the PB and the destination, and can also be significantly degraded by decreasing the channel correlation factors. We also demonstrate the effectiveness of the sensor transmit power optimization in improving the overall ergodic capacity.

## 1. Introduction

Energy harvesting (EH) is envisioned to be a promising approach for prolonging the lifetime of energy-constrained networks, typically the wireless sensor networks (WSNs) [1,2]. Traditionally, the energy is harvested from natural sources, e.g., solar, wind, heat, etc. [3]. However, this energy harvesting approach relies on some uncontrollable factors such as weather conditions, thus is not suitable to wireless communication networks that require high stability in terms of the quality-of-service (QoS). Hence, to harvest energy from the radio-frequency (RF) signals, which is capable of providing controllable energy supplies, has gained ever-increasing attention among the wireless communities [4]. An important application scenario of this method lies in wireless power transmission (WPT), which is enabled by the radio-frequency (RF) and is capable of providing convenient and continuous power supply for wireless powered devices [5]. Specifically, classic *time-switching* and *power-splitting* receiving architectures to realize practical WPT are proposed and discussed extensively in the literature [6,7,8]. To the state of the art, WPT technique has been investigated in numerous systems, e.g., cognitive radio networks [9], multiple-input–multiple-output (MIMO) networks [10], non-orthogonal multiple access (NOMA) networks [11], etc.

While the above works all assume the perfect channel state information (CSI), some recent studies have focused on the effect of imperfect CSI on the system’s energy and information transmission. In [12], the secrecy performance of a multiple-input–single-output (MISO) WPT system is studied, where the CSI used for transmit antenna selection (TAS) is outdated. In [13], the power optimization to maximize the total capacity of small cell in wireless powered heterogeneous networks is investigated, where the imperfect CSI is handled by using a non-cooperative game approach. Meanwhile, Bi and Chen et al. [14] and Liu et al. [15] introduced the Markov chain theory to formulate the dynamic behaviors of energy storage at the wireless powered devices without taking into account the imperfect CSI.

In this paper, we analyze the impact of imperfect CSI on the energy and information transmissions in the wireless powered sensor networks using the Markov chain theory. The PB supplies energy to the wireless powered sensor using MRT protocol if the energy of the sensor is not enough to conduct the transmission operation. Otherwise, the sensor transmits information and the destination receives it using MRC protocol. The differences between this work and the work in [16] are obvious. First, Tang et al. [16] considered the transmission between multiple users and a single-antenna destination, while this work studies the transmission between a single user and a multi-antenna destination. Tang et al. [16] adopted the user selection, while this work does not. Besides, this work adopts MRC at the multi-antenna destination while Tang et al. [16] did not. Moreover, this work considers the effect of imperfect CSI, while Tang et al. [16] only assumed that all the channels were perfect, which becomes a highlight of this work and makes this work much more practical.

The contributions of this paper are summarized as follows:

- To the state of the art, the proposed MRT/MRC WPT system with imperfect CSI is firstly investigated in this paper.
- The network transmit probability, and effective and overall ergodic capacities are derived under the condition of imperfect CSI during both MRT and MRC operations to assess the impact of imperfect CSI on the energy and information transmissions.
- Our results demonstrate the detrimental effect of imperfect CSI on the network transmit probability, effective and overall ergodic capacities, and the validity to improve the overall ergodic capacity by optimizing the sensor transmit power.

## 2. System Model

**Figure 1:**
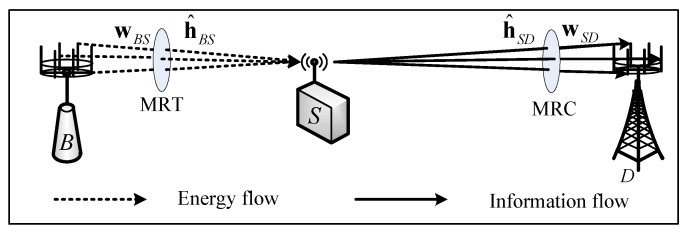
System model.

We consider a wireless powered sensor network, as shown in Figure 1, which consists of a PB node *B*, a wireless powered sensor *S*, and a destination node *D*. It is assumed that *B* and *D* are equipped with $N_{B}$ and $N_{D}$ antennas, and *S* is equipped with a single antenna. The sensor *S* is equipped with an energy storage with a finite-capacity of $\varepsilon_{T}$. We assume that all the channels experience quasi-static Rayleigh fading so that the channel coefficients keep constant during a block time $T_{0}$ but change independently from one packet time to another [7,17]. Moreover, a standard path-loss model [7] is adopted, namely the average channel power gain ${\bar{\gamma }}_{ab}=E\left[{\left|h_{ab}\right|}^{2}\right]=d_{ab}^{-\alpha }$, where $E\left[\cdot \right]$ denotes the expectation operation, α is the path-loss factor, and $h_{ab}$ and $d_{ab}$ denote the channel coefficient and the distance between *a* and *b*, respectively.

To quantify the energy storage at the sensor, we adopt a discrete-level model similar to in [14,15]. Specifically, the energy capacity of the storage is discretized into *L* units. As such, there will be 1+L possible energy levels in total at *S* with the *l*th energy level defined as

(1) $$\begin{array}{c} \varepsilon_{l}=\begin{array}{cc} l\cdot \varepsilon_{\Delta }, & l\in \left\{0,1,\cdots ,L\right\} \end{array}, \end{array}$$

where $\varepsilon_{\Delta }=\frac{\varepsilon_{T}}{L}$ is the single energy unit.

Obviously, an energy amount $P_{S}T_{0}$ is required to supply a transmission operation (note that the extra power consumption of the transmitting/receiving circuitry is neglected in this paper [7,8]), where $P_{S}$ represents the sensor transmit power. We set $P_{S}=l_{S}\frac{\varepsilon_{\Delta }}{T_{0}}$ in this paper due to the energy discretization [14], where $l_{S}$ denotes the transmit energy level corresponding to the energy consumed at *S* of each transmission with $l_{S}\in \left\{1,\cdots ,L\right\}$. We highlight that the sensor energy will always transfer within ${\left\{\varepsilon_{l}\right\}}_{l=0}^{L}$ given in Equation (1), and the transitions among energy levels form a Markov chain [14]. For the notation convenience, we denote the stationary probability vector of the Markov chain as $\pi ={\left[\pi_{0},\pi_{1},\cdots ,\pi_{l},\cdots ,\pi_{L}\right]}^{T}$, where $\pi_{l}$ is the stationary probability of the *l*th energy level $\varepsilon_{l}$.

### 2.1. Information Transmitting for $l\geq l_{S}$

When the index of the energy level at *S* satisfies $l\geq l_{S}$, the sensor energy will be sufficient so that information transmission can occur at *S*. As a result, the received signal-to-noise ratio (SNR) at *D* could be given by

(2) $$\begin{array}{c} \gamma_{D}=\gamma_{S}{\left|h_{SD}^{T}w_{SD}\right|}^{2}, \end{array}$$

where $\gamma_{S}=\frac{P_{S}}{N_{0}}$, and $N_{0}$ is the variance of the additive white Gaussian noise (AWGN). $h_{SD}={\left[h_{SD_{1}},\cdots ,h_{SD_{d}},\cdots ,h_{SD_{N_{D}}}\right]}^{T}\in C^{N_{D}\times 1}$ represents the channel coefficient vector between *S* and *D* with $d\in \left\{1,\cdots ,N_{D}\right\}$, $w_{SD}$ is the normalized MRC weight vector applied at *D* satisfying $w_{SD}=\frac{{\hat{h}}_{SD}}{∥{\hat{h}}_{SD}∥}$ due to imperfect CSI [18], and ${\hat{h}}_{SD}$ is the estimated channel coefficient vector of $h_{SD}$ that can be modeled as [12,19]

(3) $$\begin{array}{c} {\hat{h}}_{SD}=\rho_{SD}h_{SD}+\sqrt{1-\rho_{SD}^{2}}e_{SD}, \end{array}$$

where $\rho_{SD}$ denotes the channel correlation factor between the actual channel coefficient vector $h_{SD}$ and its estimation ${\hat{h}}_{SD}$, and $e_{SD}$ is the Gaussian random estimating error vector with each element having the variance of ${\bar{\gamma }}_{SD}$.

### 2.2. Information Transmitting for $l<l_{S}$

When the index of the energy level at *S* satisfies $l<l_{S}$, the energy harvesting will occur, and the harvested energy at *S* would be

(4) $$\begin{array}{c} \varepsilon_{S}=\eta T_{0}P_{B}{\left|h_{BS}^{T}w_{BS}\right|}^{2}, \end{array}$$

where $P_{B}$ is the transmit power of *B* and η denotes the energy conversion efficiency. $h_{BS}$ represents the channel coefficient vector between *B* and *S* with $b\in \left\{1,\cdots ,N_{B}\right\}$, $w_{BS}\in C^{N_{B}\times 1}$ is the normalized MRT weight vector applied at *B* satisfying $w_{BS}=\frac{{\hat{h}}_{BS}}{∥{\hat{h}}_{BS}∥}$ due to imperfect CSI, and ${\hat{h}}_{BS}$ is the estimated channel coefficient vector of $h_{BS}$ that can be modeled as [12,19]

(5) $$\begin{array}{c} {\hat{h}}_{BS}=\rho_{BS}h_{BS}+\sqrt{1-\rho_{BS}^{2}}e_{BS}, \end{array}$$

where $\rho_{BS}$ denotes the channel correlation factor between the actual channel coefficient vector $h_{BS}$ and its estimation ${\hat{h}}_{BS}$, and $e_{BS}$ is the Gaussian random estimating error vector with each element having the variance of ${\bar{\gamma }}_{BS}$. Then, the harvested energy that can be saved in the storage of *S* after energy discretization is derived as [14,15]

(6) $$\begin{array}{c} {\tilde{\varepsilon }}_{S}=\varepsilon_{l^{*}},\mathrm{with}\;l^{*}=\underset{l\in \left\{0,1,\cdots ,L\right\}}{\arg\max}\;\left\{\varepsilon_{l}:\varepsilon_{l}\leq \varepsilon_{S}\right\}. \end{array}$$

## 3. Ergodic Capacity

The ergodic capacity is defined as the expected value of the instantaneous mutual information of the received SNR [20]. By using the total probability theorem, the overall ergodic capacity of the proposed network can be calculated as

(7) $$\begin{array}{c} \bar{C}=P_{tp}{\bar{C}}_{tp}, \end{array}$$

where $P_{tp}=\sum_{l=l_{S}}^{L}\pi_{l}$ is the transmit probability of the network, and ${\bar{C}}_{tp}$ denotes the effective ergodic capacity of the network on condition that the information transmission occurs, which is defined as [19,20]

(8) $$\begin{array}{c} {\bar{C}}_{tp}=E\left[\log_{2}\left(1+\gamma_{D}\right)\right]=\frac{1}{\ln2}\int_{0}^{\infty }\frac{1-F_{\gamma_{D}}\left(x\right)}{1+x}dx. \end{array}$$

Next, we elaborate on the derivation of ${\bar{C}}_{tp}$ and $P_{tp}$, and then focus on the optimization of $P_{S}$ to maximize $\bar{C}$.

### 3.1. Derivation of Effective Ergodic Capacity ${\bar{C}}_{tp}$

Referring to ([18], Equation (48)), the cumulative distribution function (CDF) of $\gamma_{D}$ is given by

(9) $$\begin{array}{c} F_{\gamma_{D}}\left(x\right)=1-e^{-\frac{x}{\gamma_{S}{\bar{\gamma }}_{SD}}}\sum_{m=0}^{N_{D}-1}\sum_{k=0}^{m}\frac{\psi_{m}}{k!}{\left(\frac{x}{\gamma_{S}{\bar{\gamma }}_{SD}}\right)}^{k}, \end{array}$$

where $\psi_{m}=\frac{\left(N_{D}-1\right)!\rho_{SD}^{2m}{\left(1-\rho_{SD}^{2}\right)}^{N_{D}-m-1}}{\left(N_{D}-m-1\right)!m!}$, and $F_{\gamma }\left(\cdot \right)$ represents the CDF of random variable γ. Differentiating Equation (9) in the cases of k=0 and k>0, we have

(10) $$\begin{array}{c} 1-F_{\gamma_{D}}\left(x\right)=e^{-\frac{x}{\gamma_{S}{\bar{\gamma }}_{SD}}}\left[\sum_{m=0}^{N_{D}-1}\psi_{m}\right.\left.+\sum_{m=1}^{N_{D}-1}\sum_{k=1}^{m}\frac{\psi_{m}}{k!}{\left(\frac{x}{\gamma_{S}{\bar{\gamma }}_{SD}}\right)}^{k}\right]. \end{array}$$

Substituting Equation (10) into Equation (8), we derive ${\bar{C}}_{tp}$ as

(11) $$\begin{array}{c} {\bar{C}}_{tp}=\frac{1}{\ln2}\sum_{m=0}^{N_{D}-1}\psi_{m}\underset{I_{1}}{\underset{︸}{\int_{0}^{\infty }\frac{e^{-\frac{x}{\gamma_{S}{\bar{\gamma }}_{SD}}}}{1+x}dx}}+\frac{1}{\ln2}\sum_{m=1}^{N_{D}-1}\sum_{k=1}^{m}\frac{\psi_{m}}{k!}\underset{I_{2}}{\underset{︸}{\int_{0}^{\infty }\frac{e^{-\frac{x}{\gamma_{S}{\bar{\gamma }}_{SD}}}{\left(\frac{x}{\gamma_{S}{\bar{\gamma }}_{SD}}\right)}^{k}}{1+x}dx}}. \end{array}$$

Resorting to ([21], eq. (3.352.4)) and ([21], eq. (3.353.5)), we derive $I_{1}$ and $I_{2}$ as

(12) $$\begin{array}{c} I_{1}=-Ei\left(\frac{-1}{\gamma_{S}{\bar{\gamma }}_{SD}}\right)e^{\frac{1}{\gamma_{S}{\bar{\gamma }}_{SD}}}, \end{array}$$

(13) $$\begin{array}{c} I_{2}=\sum_{k_{0}=1}^{k}\left(k_{0}-1\right)!{\left(\frac{-1}{\gamma_{S}{\bar{\gamma }}_{SD}}\right)}^{k-k_{0}}-{\left(\frac{-1}{\gamma_{S}{\bar{\gamma }}_{SD}}\right)}^{k}Ei\left(\frac{-1}{\gamma_{S}{\bar{\gamma }}_{SD}}\right)e^{\frac{1}{\gamma_{S}{\bar{\gamma }}_{SD}}}. \end{array}$$

Substituting Equations (12) and (13) into Equation (11), we easily derive ${\bar{C}}_{tp}$.

### 3.2. Derivation of Transmit Probability $P_{tp}$

From the calculation expression of $P_{tp}$ given after Equation (7), we need to derive the stationary probability vector of the Markov chain π. According to the Markov chain theory, to derive π, we need to figure out the transition probabilities among all the energy levels first. Without loss of generality, we examine the transition probability from $\varepsilon_{l}$ to $\varepsilon_{l^{\prime }}$ within one transition, $l,l^{\prime }\in \left\{0,1,\cdots ,L\right\}$.

#### 3.2.1. Transition for $l\geq l_{S}$

As described in Section 2, the sensor will transmit information when $l\geq l_{S}$ so that the energy in its storage will decrease $\varepsilon_{l_{S}}$. As a result, the transition probability from $\varepsilon_{l}$ to $\varepsilon_{l^{\prime }}$ will be $P_{l,l^{\prime }}=1$ only when $\Delta l=-l_{S}$, where $\Delta l=l^{\prime }-l$.

#### 3.2.2. Transition for $l<l_{S}$

For $l<l_{S}$, the sensor will be not able to transmit information and has to harvest energy from the PB. Therefore, Δl<0 is not possible to occur because the energy saved in the storage is not possible to decrease if energy harvesting is occurred. For the case with Δl≥0 and $l^{\prime }<L$, we know that there would be an energy increment of $\varepsilon_{\Delta l}$ only when the harvested energy $\varepsilon_{S}$ satisfying $\varepsilon_{\Delta l}\leq \varepsilon_{S}<\varepsilon_{\Delta l+1}$, which results in $P_{l,l^{\prime }}=F_{\varepsilon_{S}}\left(\varepsilon_{\Delta l+1}\right)-F_{\varepsilon_{S}}\left(\varepsilon_{\Delta l}\right)$ with $\varepsilon_{S}$ given in Equation (4). On the contrary, the event of Δl≥0 and $l^{\prime }=L$ will occur as long as $\varepsilon_{S}\geq \varepsilon_{\Delta l}$, which leads to $P_{l,l^{\prime }}=1-F_{\varepsilon_{S}}\left(\varepsilon_{\Delta l}\right)$.

As such, the transition probability from $\varepsilon_{l}$ to $\varepsilon_{l^{\prime }}$ within one transition is summarized as

(14) $$P_{l,l^{\prime }}=\left\{\begin{array}{cc} 1, & l\geq l_{S},\Delta l=-l_{S},\\ F_{\varepsilon_{S}}\left(\varepsilon_{\Delta l+1}\right)-F_{\varepsilon_{S}}\left(\varepsilon_{\Delta l}\right), & l<l_{S},\Delta l\geq 0,l^{\prime }<L,\\ 1-F_{\varepsilon_{S}}\left(\varepsilon_{\Delta l}\right), & l<l_{S},\Delta l\geq 0,l^{\prime }=L,\\ 0, & \mathrm{others}. \end{array}\right.$$

We note that the CDF of $\varepsilon_{S}$ in Equation (14) could be readily derived from Equation (9) by making an appropriate replacement, i.e., $\gamma_{S}\to \eta T_{0}P_{B}$, ${\bar{\gamma }}_{SD}\to {\bar{\gamma }}_{BS}$, $N_{D}\to N_{B}$, $\rho_{SD}\to \rho_{BS}$. This can be easily concluded because the weight vector of MRC $w_{SD}$ has the similar form with the weight vector of MRT.

Denote $A\in R^{\left(L+1\right)\times \left(L+1\right)}$ as the transition matrix with its $\left(l+1,l^{\prime }+1\right)$th element being $A_{\left(l+1\right),\left(l^{\prime }+1\right)}=P_{l,l^{\prime }}$. It is easy to know that A is irreducible and row stochastic. Hence, the stationary probability vector can be derived as [14,15]

(15) $$\begin{array}{c} \pi ={\left(A^{T}-E+Q\right)}^{-1}b, \end{array}$$

where $b={\left(1,1,\cdots 1\right)}^{T}$, E is the identity matrix, and Q is an all-ones matrix. Hence, $P_{tp}=\sum_{l=l_{S}}^{L}\pi_{l}$ can be then derived.

### 3.3. Sensor Transmit Power Optimization

From the overall ergodic capacity definition in Equation (7), we see that there exists a trade-off between the value of $P_{S}$ and the overall ergodic capacity of the network. On the one hand, increasing $P_{S}$ leads to decreased $P_{tp}$, and then degrades overall ergodic capacity. On the other hand, increasing $P_{S}$ also results in increased ${\bar{C}}_{tp}$, and thus is beneficial to improve the overall ergodic capacity at the same time. As a result, there exists an optimum sensor transmit power $P_{S}^{*}$ to maximize the overall ergodic capacity of the considered network. Mathematically, the optimization of $P_{S}$ to maximize the overall ergodic capacity can be modeled as

(16) $$\begin{array}{c} \begin{array}{c} P_{S}^{*}=l_{S}^{*}\frac{\varepsilon_{\Delta }}{T_{0}}=\frac{\varepsilon_{\Delta }}{T_{0}}\arg\max_{l_{S}}\bar{C}\left(l_{S}\right),\\ \begin{array}{cc} s.t. & l_{S}\in \left\{1,2,\cdots ,L\right\}. \end{array} \end{array} \end{array}$$

Note that the closed-form expression of $P_{S}^{*}$ is intractable. As an alternative, it can be solved by applying the exhaustive method conveniently because it is an one-dimensional problem and the argument $l_{S}$ only takes finite values.

## 4. Numerical Results

In this section, we present the numerical results to illustrate the impacts of various system parameters on the performance of the proposed network. Without loss of generality, we set $P_{B}=40$ dBm, $\varepsilon_{T}=50$ mJ, L=10, η=0.8, α=3, $\rho_{BS}=\rho_{SD}=\frac{2}{3}$, $N_{B}=N_{D}=3$, $d_{BS}=10$ m, $d_{SD}=300$ m, $T_{0}=1$ s, and $N_{0}=-60$ dBm, unless otherwise stated.

**Figure 2:**
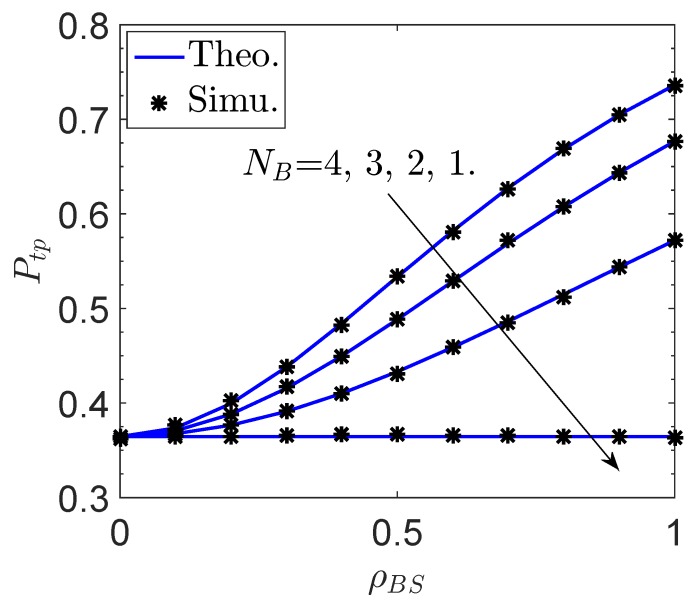
Transmit probability $P_{tp}$ of the proposed network vs. the channel correlation factor $\rho_{BS}$.

**Figure 3:**
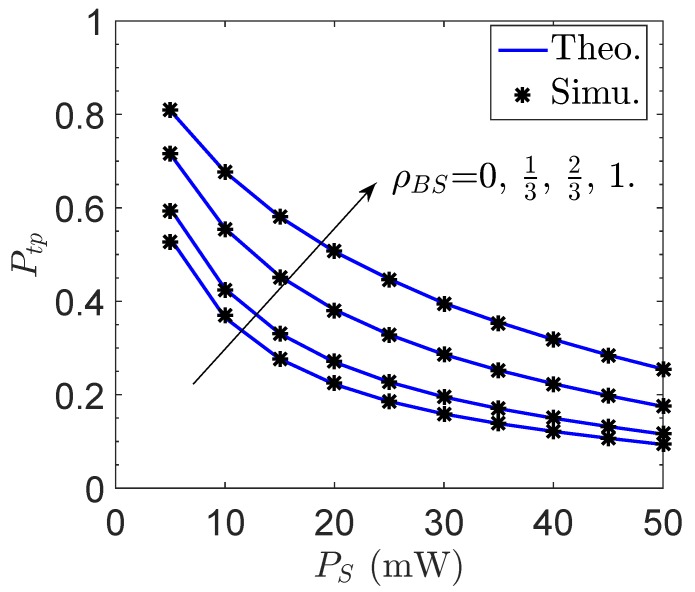
Transmit probability $P_{tp}$ of the proposed network vs. the sensor transmit power $P_{S}$.

Figure 2 and Figure 3 plot the transmit probability $P_{tp}$ of the proposed network versus the channel correlation factor between *B* and *S*, $\rho_{BS}$, and the sensor transmit power $P_{S}$, respectively. We note that $N_{B}\geq 2$ is required to conduct MRT operation, and the case of $N_{B}=1$ is presented as a benchmark when MRT is not applied. As can be expected, the energy transmission is greatly improved due to MRT with $N_{B}$ increases, so that $P_{tp}$ is largely improved. However, $P_{tp}$ will be significantly degraded with decreasing $\rho_{BS}$. Specifically, the MRT operation does not bring any benefit when $\rho_{BS}=0$ regardless of the value of $N_{B}$. This is because there is little correlation between $h_{SD}$ and ${\hat{h}}_{SD}$ with $\rho_{BS}=0$. Besides, we find from the results in Figure 3 that $P_{tp}$ will severely decrease with the increase of $P_{S}$. The reason is that a larger transmit power is generally more difficult to be satisfied for the wireless powered sensor.

**Figure 4:**
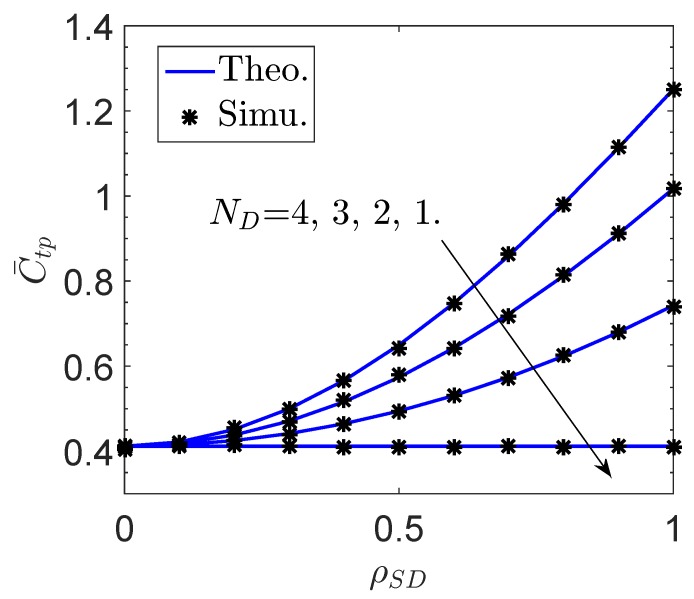
Effective ergodic capacity ${\bar{C}}_{tp}$ of the proposed network vs. the channel correlation factor $\rho_{SD}$.

**Figure 5:**
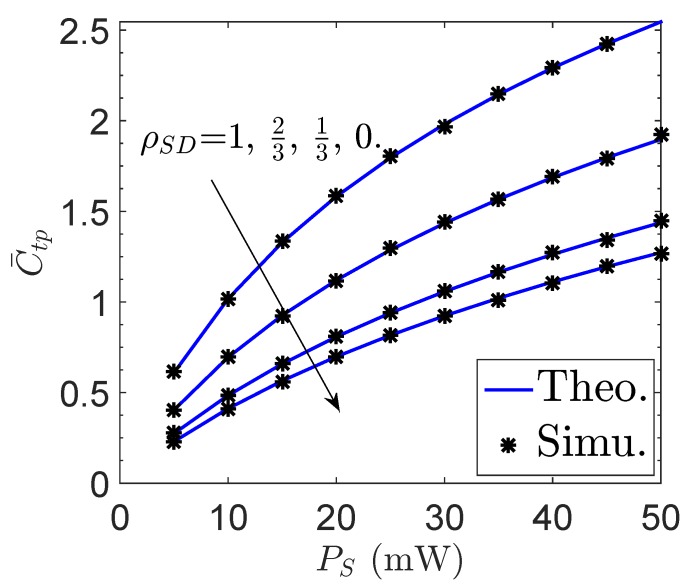
Effective ergodic capacity ${\bar{C}}_{tp}$ of the proposed network vs. the sensor transmit power $P_{S}$.

Figure 4 and Figure 5 plot the effective ergodic capacity ${\bar{C}}_{tp}$ of the proposed network versus the channel correlation factor between *S* and *D*, $\rho_{SD}$, and the sensor transmit power $P_{S}$, respectively. Similarly, the effective ergodic capacity ${\bar{C}}_{tp}$ can be enhanced by increasing the number of antennas at *D*, which however degrades with the decrease of $\rho_{SD}$. Specifically, the MRC becomes invalid when $\rho_{SD}=0$ regardless of the value of $N_{D}$. Moreover, it is observed in Figure 5 that ${\bar{C}}_{tp}$ can be improved by increasing the sensor transmit power, because a larger sensor transmit power generally leads to a greater received SNR at *D*.

**Figure 6:**
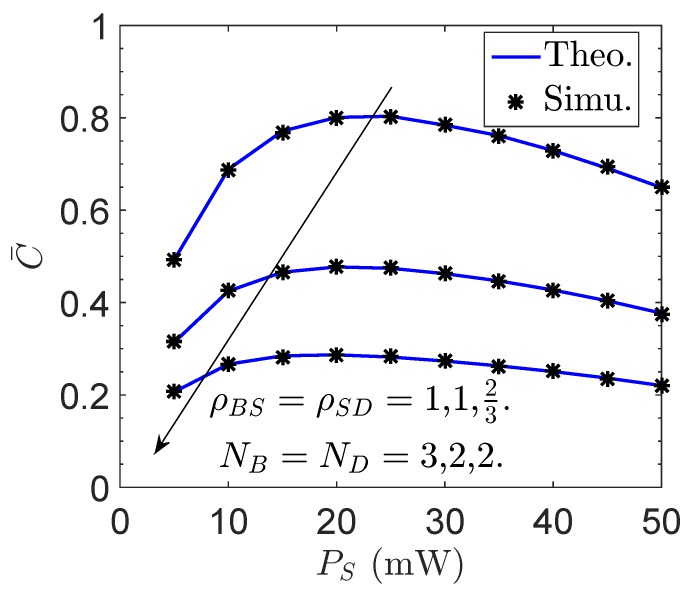
Overall ergodic capacity $\bar{C}$ of the proposed network vs. the sensor transmit power $P_{S}$.

**Figure 7:**
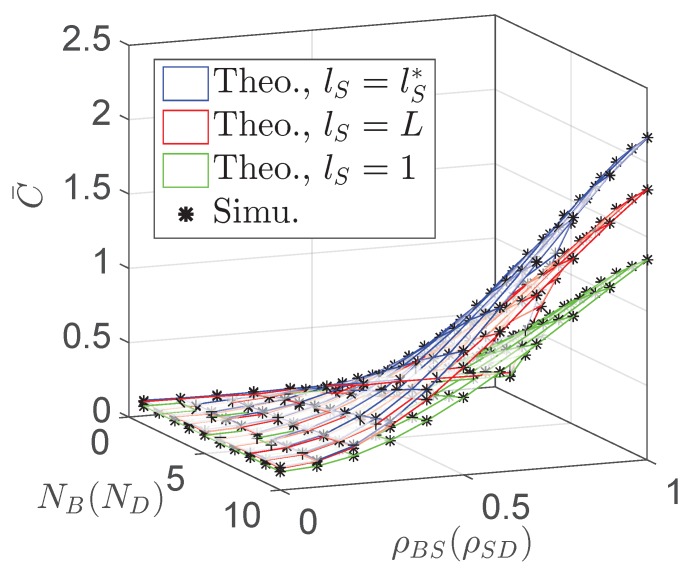
Overall ergodic capacity $\bar{C}$ of the proposed network vs. the number of antennas $N_{B}$ and the channel correlation factor $\rho_{BS}$.

Figure 6 and Figure 7 plot the overall ergodic capacity $\bar{C}$ of the proposed network versus the sensor transmit power $P_{S}$, the number of antennas at *B*, $N_{B}$, and the channel correlation factor between *B* and *S*, $\rho_{BS}$, respectively. In Figure 6, a trade-off between the value of $P_{S}$ and ergodic capacity is observed for various $N_{B}$ and $\rho_{BS}$. In addition, Figure 7 presents the maximized overall ergodic capacity $\bar{C}$ with different sensor transmit power. Note that, in this figure, $l_{S}=1$ and $l_{S}=L$ correspond to $P_{S}=5$ mW and $P_{S}=50$ mW, respectively. As can be seen, the overall ergodic capacity can be greatly improved with the proposed sensor transmit power optimization.

## 5. Application and Future Work

WPT technique can have potential applications in various scenarios of low power devices. Promising applications include health-care monitoring by implantable bio-medical sensors, architectural structure monitoring using embedding sensors in bridges, building, roads, etc. Besides, this technique can also be widely applied in the emerging construction of smart city, where numerous home-based low power wireless devices can be effectively and conveniently powered. For example, in 2017, the PB-based product “Cota Tile” was designed by Ossia Inc. to charge wireless devices at home, which received the “Innovation Awards” at the 2017 Consumer Electronics Show (CES) [22]. However, WPT technique also imposes various challenging issues to be addressed before it becomes a key technology for the future communications systems. For example, hardware development is greatly needed so that the harvesting circuits can obtain as much energy as possible. Besides, communication and energy security must be carefully considered in such systems. Overall, for the successful application of WPT systems, numerous challenges must be tackled at a cross-layer perspective from hardware implementation to specific architectural design.

## 6. Conclusions

PB assisted information and energy transmissions in the wireless powered sensor networks were studied taking into account the imperfect CSI. The energy arrival and departure of the finite-capacity storage at the sensor were characterized by adopting the Markov chain theory, and the analytical expressions of the network transmit probability, and effective and overall ergodic capacities were formulated and obtained. In addition, the sensor transmit power optimization to maximize the overall ergodic capacity was solved. The results indicate that the network transmit probability and the effective ergodic capacity could be greatly improved by increasing the number of antennas at the PB and the destination, respectively. Moreover, it was depicted that the transmit probability and the effective ergodic capacity would be severely degraded by decreasing the channel correlation factors. The findings also illustrate the validity of optimizing the sensor transmit power to improve the overall ergodic capacity of the proposed network.

## Abbreviations

The following abbreviations are used in this manuscript:

AWGN	Additive white Gaussian noise
CDF	Cumulative distribution function
CSI	Channel state information
EH	Energy harvesting
MISO	Multiple-input-single-output
MRC	Maximal ratio combining
MRT	Maximal ratio transmission
PB	Power beacon
RF	Radio-frequency
TAS	Transmit antenna selection
WPT	Wireless power transmission
WSN	wireless sensor networks
